# Supplementary material for: An Explainable Machine-Learning Model for Compensatory Reserve Measurement: Methods for Feature Selection and the Effects of Subject Variability
Source: Bioengineering (Basel). 2023 May 19;10(5):612. doi: 10.3390/bioengineering10050612 (PMC10215239; doi:10.3390/bioengineering10050612)
Supplement: Supplementary file 1 [file bioengineering-10-00612-s001.zip › bioengineering-2350021-supplementary.pdf]

Supplementary Information

Supplementary Table S1. Summary of all 54 extracted features with their abbreviated name and description of their calculation. Features calculations were based on [18] and [21].

Abbreviation	Description
PPI	Peak to peak interval
HRIP	Half-rise to inflection point
SBP	Systolic blood pressure
DBP	Diastolic blood pressure
PP	Pulse pressure
PA	Pulse Area
SI	Shock Index
t_sys_rise	Duration of the systolic rise
t_sys_dec	Duration of the systolic decay
t_dec	Duration of the total decay
t_sys	Duration of the systolic rise and systolic decay
t_dia	Duration of the diastolic rise and decay.
dn_sys	Difference between the pressure at the systolic peak and the first inflection point.
avg_sys	Average pressure of the systolic phase
avg_dia	Average pressure of the diastolic phase.
avg_sys_rise	Average pressure of the systolic rise
avg_sys_dec	Average pressure of the systolic decay
avg_dec	Average pressure of the overall decay of the waveform. From Point C to next Point A.
avg_sys_nodia	Average pressure of the systolic phase with the pressure of the diastolic pressure subtracted
avg_dia_nodia	Average pressure of the diastolic phase with the pressure of the diastolic pressure subtracted
avg_sys_rise_nodia	Average pressure of the systolic rise with the pressure of the diastolic pressure subtracted
avg_sys_dec_nodia	Average pressure of the systolic decay with the pressure of the diastolic pressure subtracted
avg_dec_nodia	Average pressure of the overall decay of the waveform with the pressure of the diastolic pressure subtracted
sys_area	Area under the curve of the systolic phase.
pp_area_nor	Area under the curve normalized by the number of samples in the waveform.

sys_area_norm	Area under the curve of the systolic phase normalized by the number of samples in the entire waveform
sys_rise_area	Area under the curve of the systolic rise.
sys_rise_area_nor	Area from the start of the beat to the systolic maximum normalized by the number of samples in the systolic rise
sys_rise_area_norm	Area from the start of the beat to the systolic maximum normalized by the number of samples of the entire waveform
sys_dec_area	Area under the curve of the systolic decay. From the systolic peak to first inflection point.
sys_dec_area_nor	Area under the curve of the systolic decay normalized by the samples in the systolic decay.
sys_dec_area_norm	Area under the curve of the systolic decay normalized by the samples in the entire waveform.
dec_area	Area under the curve of the entire waveform decay. From the systolic maximum to the start of the next beat.
dec_area_nor	Area under the curve of the entire waveform decay normalized by the number of samples in the waveform decay
dec_area_norm	Area under the curve of the entire waveform decay normalized by the number of samples in entire waveform.
dia_area	Area under the curve of the diastolic portion of the waveform
dia_area_nor	Area under the curve of the diastolic phase of the waveform normalized by the number of samples in the diastolic phase.
dia_area_norm	Area under the curve of the diastolic phase of the waveform normalized by the number of samples in the entire waveform
pp_area_nodia	Area under the beat subtracted with the pressure at the first inflection point normalized by the number of samples.
sys_area_nodia	Area under the curve of the systolic portion of the waveform subtracted by the pressure of the first inflection point.
sys_area_nor_nodia	Area under the curve of the systolic portion of the waveform subtracted by the pressure of the first inflection point normalized by the number of samples in the systolic phase
sys_area_norm_nodia	Area under the curve of the systolic portion of the waveform subtracted by the pressure of the first inflection point normalized by the number of samples in the entire waveform.
sys_rise_area_nodia	Area under the curve of the systolic rise subtracted by the pressure of the first inflection point
sys_rise_area_nor_nodia	Area under the curve of the systolic rise subtracted by the pressure of the first inflection point normalized by the number of samples in the systolic rise phase.

sys_rise_area_norm_nodia	Area under the curve of the systolic rise subtracted by the pressure of the first inflection point normalized by the number of samples in the systolic rise phase.
dec_area_nodia	Area from the systolic maximum to the start of the next beat with subtracted diastolic pressure
dec_area_nor_nodia	Area from the systolic maximum to the start of the next beat subtracted by the pressure at the first inflection point and normalized by the number of samples in the systolic decay.
dec_area_norm_nodia	Area from the systolic maximum to the start of the next beat subtracted by the pressure at the first inflection point and normalized by the number of samples in the entire waveform
dia_area_nodia	Area under the diastolic portion of the waveform with subtracted by the inflection point pressure
dia_area_nor_nodia	Area under the diastolic portion of the waveform with subtracted by the inflection point pressure normalized by the number of samples in the diastolic phase
dia_area_norm_nodia	Area under the diastolic portion of the waveform with subtracted by the inflection point pressure normalized by the number of samples in the entire waveform
slope_dia	Average slope of the diastolic phase
slope_sys	Average slope of the systolic phase
slope_desc_sys	Average slope of the systolic decay
